# Complete Genome Sequences of Streptococcus suis Pig-Pathogenic Strains 10, 13-00283-02, and 16085/3b

**DOI:** 10.1128/MRA.01137-20

**Published:** 2021-01-14

**Authors:** Boyke Bunk, Beata Jakóbczak, Volker Florian, Denise Dittmar, Ulrike Mäder, Michael Jarek, Susanne Häußler, Christoph Georg Baums, Uwe Völker, Stephan Michalik

**Affiliations:** a Leibniz Institute-German Collection of Microorganisms and Cell Cultures GmbH, Braunschweig, Germany; b Ceva Innovation Center GmbH, Dessau-Rosslau, Germany; c Department of Functional Genomics, Interfaculty Institute for Genetics and Functional Genomics, University Medicine Greifswald, Greifswald, Germany; d Helmholtz Centre for Infection Research, Braunschweig, Germany; e Institute of Bacteriology and Mycology, Centre for Infectious Diseases, Faculty of Veterinary Medicine, Leipzig University, Leipzig, Germany; University of Maryland School of Medicine

## Abstract

Streptococcus suis is an important pathogen of pigs that, as a zoonotic agent, can also cause severe disease in humans, including meningitis, endocarditis, and septicemia. We report complete and annotated genomes of S. suis strains 10, 13-00283-02, and 16085/3b, which represent the highly prevalent serotypes *cps2*, *cps7*, and *cps9*, respectively.

## ANNOUNCEMENT

The Gram-positive bacterium Streptococcus suis can infect pigs and may lead to meningitis, septicemia, polyarthritis, or endocarditis and thus large economic losses in swine farming worldwide (1). Here, we announce the complete genomes of three Streptococcus suis strains, namely, 10 (2), 13-00283-02 (3), and 16085/3b (4), which represent different important pathotypes of S. suis that have been extensively used in experimental studies with pigs (2–6).

All three strains were subjected to S. suis multiplex PCR (MP-PCR) (7) and matrix-assisted laser desorption ionization–time of flight mass spectrometry (MALDI-TOF MS) analyses for taxonomic identification. The S. suis strains were grown aerobically for 16 h at 37°C in Todd-Hewitt broth (THB) inoculated with seven colonies each from Columbia agar plates. During the exponential growth phase, 10 ml of culture was harvested by centrifugation (8,230 × *g* for 10 min at room temperature). Genomic DNA was isolated using the Qiagen Genomic-tip 100/G and the Qiagen genomic DNA buffer set according to the manufacturer’s instructions and was subjected to long-read sequencing on the PacBio RS II system at Eurofins GATC Biotech. Libraries for the PacBio sequencing were generated using the SMRTbell template preparation kit v1.0 (Pacific Biosciences). Briefly, 7.5 μg DNA was sheared by needle shearing, and 5 μg sheared DNA was end repaired and ligated overnight to hairpin adapters using the DNA/polymerase binding kit P6 v2 (Pacific Biosciences). BluePippin size selection was performed with a length cutoff value of 10 kb. Conditions for annealing of sequencing primers and binding of polymerase to a purified SMRTbell template were assessed with the BindingCalculator in RS Remote. Single-molecule real-time (SMRT) sequencing was carried out on the PacBio RS II system, recording one 240-min movie per SMRT cell, which resulted in 66,047, 95,957, and 66,304 postfiltered reads for strains 10, 13-00283-02, and 16085/3b, respectively, with mean read lengths of 12,864 bp (*N*_50_, 17,849 bp), 12,513 bp (*N*_50_, 16,957 bp), and 13,190 bp (*N*_50_, 16,957 bp), respectively. Libraries for Illumina sequencing were prepared using a NEBNext Ultra II FS DNA library preparation kit, and 300-bp size selection was performed using magnetic beads. Short-read sequences were obtained on a MiSeq system (Illumina, San Diego, CA, USA), leading to 301,073, 237,363, and 277,542 reads of 2 × 301 bp for strains 10, 13-00283-02, and 16085/3b, respectively. Quality control was performed using FastQC (https://www.bioinformatics.babraham.ac.uk/projects/fastqc). Total numbers of 367,120, 333,320, and 343,558 reads were generated for strains 10, 13-00283-02, and 16085/3b, respectively.

Long-read genome assembly was performed with the RS HGAP Assembly 3 protocol v2.3.0, applying a minimum subread length of 500 bp and a minimum polymerase read quality score of 0.8. Chromosomes were fully assembled in all three cases. Additional contigs could be excluded by identifying them as assembly artifacts contained within the chromosomal contig. All chromosomal contigs were circularized, artificial redundancies at the ends of the contigs were removed, and the chromosomes were adjusted to *dnaA* as the first gene. Identification of redundancies and *dnaA* was carried out by BLAST, and circularization and rotation were performed using the genomecirculator.jar tool (https://github.com/boykebunk/genomefinish). Error correction was performed by mapping the short reads onto long-read assembled genomes using Burrows-Wheeler alignment with BWA v0.6.2 in paired-end mode with default settings (8), with subsequent variant and consensus calling using VarScan v2.3.6 (9). Genome annotation was based on Prokka v1.14.6 (10). The sequencing and annotation results are summarized in Table 1. The sequencing summary statistics were generated with SAMtools v1.10 by mapping the raw Illumina reads using Bowtie 2 v2.3.5 and the PacBio subreads using BWA-MEM v0.7.17-r1188 to the final completely assembled genomes.

**TABLE 1:** Sequencing and annotation results

Sequencing method or annotation	Data for S. suis strain:		
	10	13-00283-02	16085/3b
PacBio sequencing (mapped reads)			
No. of subreads	106,390	130,467	103,650
Avg read length (bp)	7,960	9,183	8,409
Avg quality score	9.8	9.9	9.8
Sequencing throughput (bp)	8.47E+08	1.20E+09	8.72E+08
Avg coverage depth (×)	346	424	331
Genome coverage (%)	100	100	100
SRA accession no.	SRR11931227	SRR11931225	SRR11931223
Illumina sequencing (mapped reads)			
No. of reads	602,146	474,726	555,084
Avg read length (bp)	301	301	301
Avg quality score	35.3	35.0	35.2
Sequencing throughput (bp)	1.81E+08	1.43E+08	1.67E+08
Avg coverage depth (×)	85	63	70
Genome coverage (%)	100	100	99.9988
SRA accession no.	SRR11931228	SRR11931226	SRR11931224
Prokka v1.14 annotation			
GenBank accession no.	CP058742	CP058741	CP058740
Genome length (bp)	2,042,887	2,182,685	2,172,677
No. of rRNAs	12	12	12
No. of tRNAs	56	57	56
No. of coding sequences	1,945	2,099	2,069
No. of genes	2,014	2,169	2,138
GC content (%)	41.3	41.1	41.1

The fully assembled genomes range from 2,042,887 bp (strain 10) to 2,172,677 bp (strain 16085/3b) to 2,182,685 bp (strain 13-00283-02), with GC contents of 41.1 to 41.3%. The complete and annotated sequences of the pathogenic S. suis strains reported here are a prerequisite for advanced basic research and therapy development.

### Data availability.

PacBio and Illumina data and the annotated genomes are available under BioProject accession number PRJNA637499 and the accession numbers listed in Table 1.
